# Regulatory T cells (Tregs) in lymphoid malignancies and the impact of novel therapies

**DOI:** 10.3389/fimmu.2022.943354

**Published:** 2022-08-01

**Authors:** Kamira Maharaj, Angimar Uriepero, Eva Sahakian, Javier Pinilla-Ibarz

**Affiliations:** ^1^ Department of Immunology, H. Lee Moffitt Cancer Center & Research Institute, Tampa, FL, United States; ^2^ Department of Malignant Hematology, H. Lee Moffitt Cancer Center & Research Institute, Tampa, FL, United States

**Keywords:** Treg, B cell, non-Hodgkin’s lymphoma, chronic lymphocytic leukemia, immune checkpoint blockade, BTK, PI3K, BCL-2

## Abstract

Regulatory T cells (Tregs) are responsible for maintaining immune homeostasis by controlling immune responses. They can be characterized by concomitant expression of FoxP3, CD25 and inhibitory receptors such as PD-1 and CTLA-4. Tregs are key players in preventing autoimmunity and are dysregulated in cancer, where they facilitate tumor immune escape. B-cell lymphoid malignancies are a group of diseases with heterogenous molecular characteristics and clinical course. Treg levels are increased in patients with B-cell lymphoid malignancies and correlate with clinical outcomes. In this review, we discuss studies investigating Treg immunobiology in B-cell lymphoid malignancies, focusing on clinical correlations, mechanisms of accumulation, phenotype, and function. Overarching trends suggest that Tregs can be induced directly by tumor cells and recruited to the tumor microenvironment where they suppress antitumor immunity to facilitate disease progression. Further, we highlight studies showing that Tregs can be modulated by novel therapeutic agents such as immune checkpoint blockade and targeted therapies. Treg disruption by novel therapeutics may beneficially restore immune competence but has been associated with occurrence of adverse events. Strategies to achieve balance between these two outcomes will be paramount in the future to improve therapeutic efficacy and safety.

## Introduction

Lymphoid malignancies are a diverse spectrum of diseases including malignancies arising from transformation of B cells, T cells or natural killer (NK) cells (1). Lymphoid malignancies are systemic diseases that can manifest in lymph nodes, bone marrow and peripheral blood. They are among the most common cancers and one of the leading causes of cancer-related death in the United States (2). B-cell non-Hodgkin’s lymphoma (B-NHL) accounts for the majority of lymphoid malignancies and is classified as indolent [grade I/II follicular lymphoma (FL), small lymphocytic lymphoma (SLL), marginal zone lymphoma (MZL)] or aggressive [diffuse large B-cell lymphoma (DLBCL), Burkitt lymphoma (BL)]. SLL cells are immunophenotypically identical to chronic lymphocytic leukemia (CLL) cells, but the patients do not present with lymphocytosis (3). Therefore, both clinical presentations are frequently referred to as the same disease. Although CLL/SLL belongs to the indolent lymphoma group, approximately 3% of patients develop Richter syndrome, undergoing transformation toward aggressive DLBCL (4, 5).

Since the approval of the first anti-CD20 monoclonal antibody (mAb) more than twenty years ago, chemoimmunotherapy has been utilized for first-line treatment of lymphoid malignancies. Rituximab, an anti-CD20 mAb, is often used as monotherapy in grade I/II follicular lymphoma and elicits high overall response (6). First-line treatment with fludarabine, cyclophosphamide and rituximab (FCR) is still an option for CLL patients with mutated immunoglobulin heavy chain gene (IgHV) which is associated with better prognosis (3). However, treatment refractoriness and low rates of response to chemoimmunotherapy are frequently seen in patients with aggressive lymphomas such as DLBCL (7, 8). Targeted therapies such as B-cell receptor (BCR) signaling inhibitors have improved survival of patients with lymphoid malignancies (9–11), but long-term remission is lacking in a subset of patients and most require prolonged treatment (12). Immune checkpoint blockade (ICB) is currently being investigated for use in B-cell malignancies but has produced underwhelming results thus far with the exception of Hodgkin’s disease and mediastinal DLBCL (13). Chimeric antigen receptor (CAR) T-cell therapies has become an option in the third-line setting for patients with B-NHL and has also been recently approved in the second line for DLBCL (14, 15). Despite availability of novel therapeutics, relapsed/refractory (R/R) disease continues to be an unmet need and mechanisms underlying response to treatment need to be further explored.

Immune cell subsets are altered within the tumor microenvironment (TME) of B-NHL and are implicated in malignant cell survival and drug resistance (16). Alterations in T-cell phenotype and function have been broadly observed (17–20). In the FL TME, malignant B cells depend on direct contact with T follicular helper (Tfh) cells, which are essential for the formation and maintenance of the germinal center reaction (21, 22). In CLL and DLBCL, investigators have observed altered CD4^+^ to CD8^+^ T cell ratio, inverted T helper (Th)1 to Th2 ratio and increased exhausted T cells expressing TIM-3, LAG-3 and PD-1 (23, 24). T cells from patients with FL, DLBCL and CLL also show impaired immune synapse formation which has been identified as an active immunosuppressive mechanism (19, 20).

Regulatory T cells (Tregs) are conventionally characterized as CD4^+^ T cells constitutively expressing high surface CD25 (IL-2Rα), low or no CD127 (IL-7Rα) and the transcription factor FoxP3 (25). Tregs are divided into two broad groups based on origin: thymic-derived natural (n)Tregs and peripherally-derived inducible (i)Tregs (26). They are essential for maintaining peripheral self-tolerance and preventing autoimmunity, and loss of the *Foxp3* gene results in multiorgan autoimmune disorder in humans and mice (27, 28). Tregs suppress activity of other immune subsets including conventional and effector T cells, B cells, NK cells, NKT cells, monocytes and dendritic cells. Numerous mechanisms of Treg-mediated suppression have been described, including (1) production of immunosuppressive cytokines such as IL-10, TGF-β and IL-35, (2) cytotoxicity by granzymes and perforin, (3) expression of co-inhibitory molecules such as PD-1, CTLA-4, and LAG-3, and (4) interference with metabolism *via* IL-2 deprivation, adenosine production and cAMP transfer (29, 30).

Tregs are dysregulated in the TME and are key players in tumor immune escape (31). Tregs have been associated with poor prognoses in patients with solid tumors and are thought to exert tumor-promoting roles by directly interfering with cytotoxic T-cell function to suppress antitumor immune responses (31). However, Tregs have been paradoxically correlated with both positive and negative clinical outcomes in B-NHL patients (32). The impact of novel therapies on Treg function, and the role of Tregs in response to therapy and incidence of adverse events have recently become relevant. In this review, we summarize and discuss studies exploring Treg immunobiology in B-NHL, focusing primarily on but not limited to FL, DLBCL and CLL.

## Prognostic value

Tregs associate with clinical outcomes in patients with lymphoid malignancies. In the following studies of FL and DLBCL patients, Tregs were detected by immunohistochemistry in biopsy specimens and identified as FoxP3^+^ unless otherwise indicated. Increased tumor-infiltrating Tregs correlated with better response to therapy and improved overall survival (33), good prognosis defined by follicular lymphoma international prognostic index (FLIPI) score (34), disease-specific and failure-free survival in FL patients (35). Two independent studies found that a diffuse distribution pattern of Tregs in FL specimens correlated with more favorable outcomes than intrafollicular or perifollicular patterns (36, 37).

Other studies similarly reported that tumor-infiltrating Tregs positively associated with overall survival in untreated DLBCL patients (38) and in DLBCL patients treated with chemoimmunotherapy (39–41). Tregs have been examined by flow cytometry in peripheral blood mononuclear cells (PBMCs) of newly diagnosed and treated DLBCL patients (42). Results revealed elevated Tregs present in newly diagnosed patients and patients at remission compared to those with R/R disease (42). Meta-analysis of 23 prior studies including but not limited to B-NHL patients revealed that higher density of tumor-infiltrating Tregs correlated with improved progression-free and overall survival (43). One conflicting report showed that Tregs associated with inferior overall survival in a cohort of DLBCL patients treated with chemoimmunotherapy, but these results were not replicated in a second cohort (23). The overall trend suggests that higher Treg levels correlate with better outcomes in FL and DLBCL patients.

The studies mentioned here examined Tregs by flow cytometry in PBMCs of CLL patients. Elevated Treg levels have been described in untreated CLL patients compared to treated CLL patients and healthy donors (44). However, increased Tregs in CLL patients correlated with worse prognostic factors including Rai and Binet stage, CD38 expression, lactate dehydrogenase (LDH) levels (45–47), and shorter time to first treatment (48, 49). Skewed Treg to Th17 ratio that favored Tregs also correlated with advanced disease (50). None of the studies reviewed here reported association of Tregs with survival, IgHV mutation status or ZAP70 expression in CLL patients. The overall trend therefore suggests that higher Treg levels indicated worse outcomes in CLL patients. Treg associations with clinical outcomes in B-NHL and CLL patients have been summarized in **Table 1**.

**Table 1:** Prognostic value of Tregs in patients with B-cell lymphoid malignancies.

Disease	Treg alteration	Correlations with outcomes	Reference
FL	increased	better response to therapy, improved overall survival	(33)
FL	increased	lower FLIPI score	(34)
FL	increased	improved disease-specific survival, improved failure-free survival	(35)
FL	diffuse distribution pattern	improved overall survival, improved progression-free survival, lower risk of transformation	(36)
FL	diffuse distribution pattern	improved survival	(37)
DLBCL	increased	improved disease-specific survival	(35)
DLBCL	increased	improved overall survival	(38–41)
DLBCL	increased	inferior overall survival	(23)
CLL	increased	higher Rai stage, CD38 expression, LDH levels	(45)
CLL	increased	higher Binet stage	(46, 47)
CLL	increased	shorter time to first treatment	(48, 49)
B-NHL	increased	improved progression-free survival, improved overall survival	(43)

### Induction and recruitment to the tumor microenvironment

**Figure 1:**
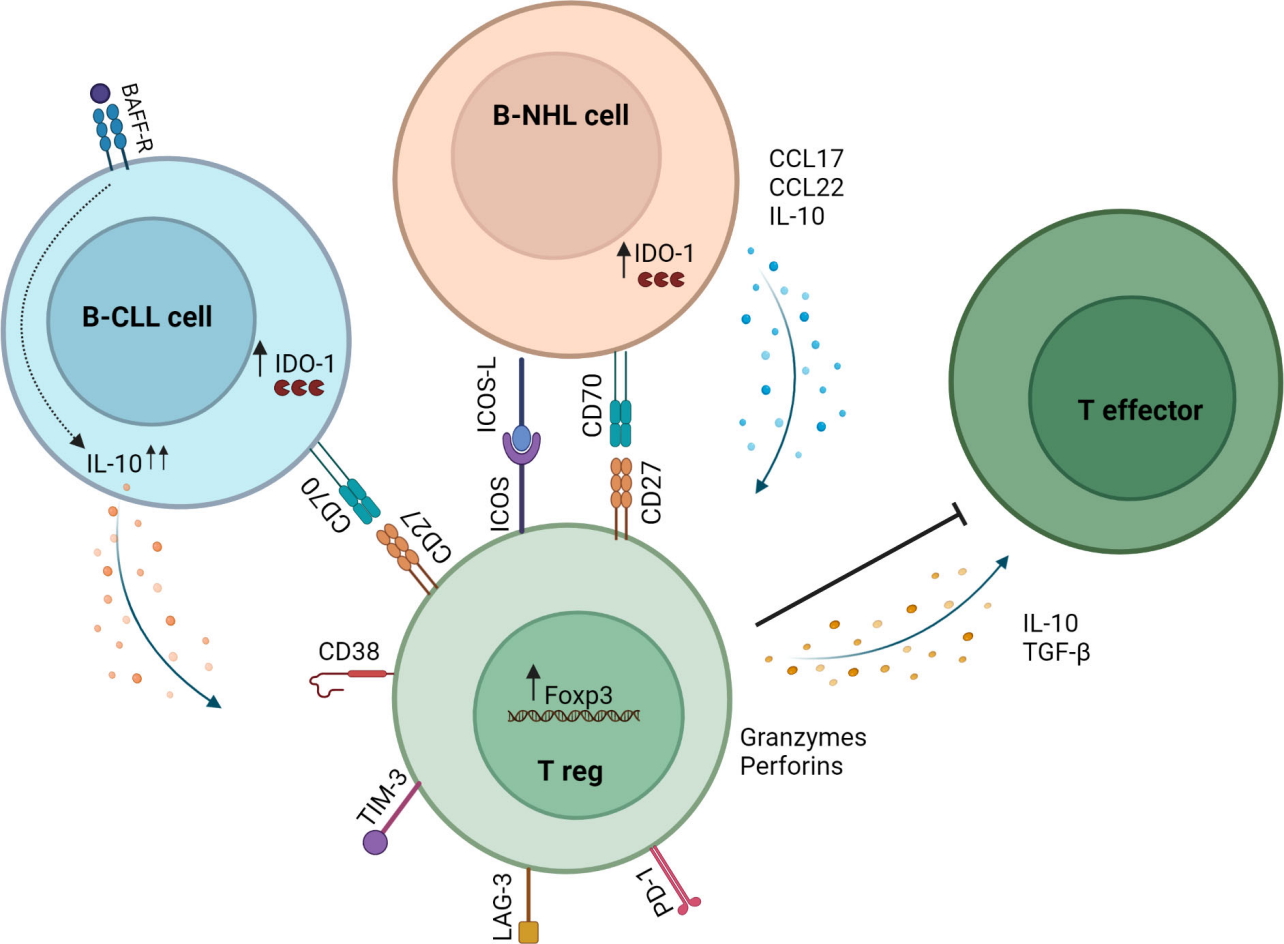
Mechanisms of Treg induction and action in the B-NHL microenvironment. B-NHL and CLL cells can induce Tregs *via* direct contact with T cells in the tumor microenvironment and through secretion of soluble factors. In B-NHL and CLL, Tregs can inhibit the immune response through the secretion of IL-10 and TGF-β or by killing effector T cells *via* release of granzymes and perforins. Tumor-infiltrating Tregs in B-NHL and CLL also exhibit high expression of immune checkpoint molecules such as PD-1, LAG-3 and TIM-3.

Mechanisms that increase Tregs in lymphoid malignancies have been widely investigated. Reports indicate that malignant cells directly induce Tregs and that proteins dysregulated by malignant cells facilitate Treg induction (**Figure 1**). B-NHL cells isolated from patients induced functional Tregs from naïve T cells in a contact-dependent manner (51). Yang et al. showed that CD70^+^ B-NHL cells induced FoxP3 expression in naïve T cells, and this was abrogated by CD70-CD27 axis blockade (52). Years later, Balsas et al. established that CD70 upregulation in SOX11^+^ MCL could promote tumor immune evasion *via* Treg induction (53). Another study revealed a role for IDO1 in B-NHL-mediated Treg induction (54). IDO1 expression positively correlated with FoxP3 expression in B-NHL, and IDO1 inhibition attenuated B-NHL-mediated Treg induction (54). The ICOS-ICOSL axis has been shown to promote FL-mediated Treg induction (55). Results demonstrated that ICOS^+^ Tregs were induced by coculture with ICOSL^+^ FL cells, and this was abrogated by ICOS-ICOSL blockade.

CLL cells produce IL-10 and share features with normal regulatory B cells referred to as Breg or B10 (56). BAFF is a protein implicated in development and maintenance of CLL cells (57). One study uncovered a key role for BAFF-driven IL-10 production by CLL cells in generating Tregs and enhancing suppressive capacity by upregulating FoxP3 expression (58). Another study demonstrated involvement of IL-10 and TGF-β1 in CLL-mediated induction of Tregs (59). Results showed that CLL cells induced Tregs from naïve T cells, and this was abrogated by blocking IL-10 or TGF-β1 (59). These studies highlight the role of soluble factors in promoting immunosuppression *via* Treg induction in the CLL microenvironment.

It has been hypothesized that circulating Tregs can be preferentially recruited into the lymphoma microenvironment. A mechanism whereby Tfh cells induce CCL17 and CCL22 production by FL cells to facilitate Treg recruitment has been elucidated (60). In another study, gene expression analysis of Tregs sorted from FL patients revealed a mechanism by which Tregs target to and accumulate within the TME through downregulation of S1PR1, SELL (L-selectin) and CCR7 (61). These data suggest that migratory capacity can be influenced by tumor cells and thus contribute to Treg accumulation in the TME.

Besides malignant cells, other cell subsets within the TME influence Treg biology. Myeloid-derived suppressor cells (MDSCs), which are increased in CLL patient PBMCs, express high levels of IDO and correlate with Treg frequency (62). MDSCs generated from CLL patient samples induced Tregs in coculture experiments. Further, the presence of MDSCs coculture enhanced CLL-mediated Treg induction (62). Collectively, these studies offer mechanistic insight into how lymphoma and CLL cells reshape the TME. Tregs can be directly induced from naïve T cells by tumor cells, or preferentially recruited and retained to the TME. Blocking antibodies or antagonists to inhibit Treg induction and recruitment to shift the balance in favor of antitumor immunity should be further investigated.

## Phenotypic and functional complexity

Tregs consist of phenotypically diverse subsets and exhibit specialized functions in lymphoid and non-lymphoid peripheral tissue (63). This diversity suggests that Tregs can alter homeostatic, migratory and suppression programs according to environmental cues (25). Investigators seeking to address the role of Tregs in lymphoma and CLL have described phenotypic and functional complexity. The studies discussed in this section indicate that Tregs harbor altered surface markers and gene expression. Tregs express variable CD25 and CD127 levels, memory, activation, Th and cytotoxicity markers. The heterogeneity of these findings might depend on which Treg subsets are present and influenced by immunoediting effects of tumor cells and microenvironmental cues. Although reports suggest diverse phenotype and function for Tregs, most studies reviewed here suggest that Tregs protect tumor cells by suppressing antitumor T-cell responses.

### Tumor-promoting Tregs

Some evidence suggests that Tregs permit tumor immune evasion in B-NHL. Studies have shown that B-NHL-derived Tregs suppressed T-cell proliferation and IFN-γ production, indicating a tumor-protective effect mediated by Tregs (64, 65). Tumor-promoting Tregs in CLL appear to be resistant to apoptosis and exhibit sustained suppressive ability. Both conventional CD4^+^CD25^hi^CD127^-/lo^ and unconventional CD4^+^CD25^-/lo^CD127^-^ FoxP3^+^ Tregs exhibited lower intrinsic apoptotic rates in CLL patients versus controls (47). Further, expression of anti-apoptotic protein BCL-2 in CLL T cells was associated with an immunosuppressive TME through promotion of Tregs (66). Results of this study revealed that BCL-2^+^ Tregs harbored increased expression of IL-10 and TGF-β compared to BCL-2^-^ Tregs, indicating superior suppressive capacity (66). These findings suggest that CLL Tregs may be directly targeted by venetoclax, a BCL-2 inhibitor which is approved for treatment of CLL (67).

Mouse models have proven useful in interrogating Treg phenotype and function during leukemia development in ways that are not possible in studies that solely utilize patient samples. Gorgun et al. first noted increased CD4^+^CD25^+^FoxP3^+^CTLA-4^+^ Tregs occurring alongside leukemia development in the Eμ-TCL1 transgenic mouse model of CLL (68). A comprehensive recent study established a supportive role for Tregs in CLL progression in the adoptive transfer Eμ-TCL1 mouse model (69). In this model, FoxP3^+^ Treg depletion resulted in expansion of CD8^+^ effector cells and leukemia clearance. A CD69^hi^LAG-3^hi^CD44^lo^CD25^lo^ Treg subset was identified *via* unbiased clustering analysis of flow cytometry data. This Treg subset exhibited increased expression of genes related to suppression, checkpoints, and chemokines that most likely support leukemia progression. Using the TCL1-ovalbumin (OVA)-expressing mouse model to generate T cells specific for OVA antigen expressed by leukemic cells, this study further demonstrated that Tregs derived from CLL-bearing mice could suppress proliferation of CLL-specific CD8^+^ T cells (69). Interestingly, these results reflected another study showing that elevated Treg levels in CLL patients correlated with decreased T-cell responses against viral and tumor antigens in functional assays (70). A study in the λ-MYC mouse model of BL similarly demonstrated that Tregs suppressed antitumor immune responses, and Treg depletion delayed disease development (71). Further, Tregs in this model recognized antigens likely to be displayed by tumor cells (71). These reports provide evidence that Tregs can potentiate immune evasion in an antigen-specific manner.

### Tregs harboring effector and unconventional phenotypes

Tregs expressing effector T-cell characteristics have been identified in lymphoma and CLL patients. However, there is no standard method of identifying effector-like Tregs at this point. Tregs harboring cytotoxic capacity, expression of Th lineage specific transcription factors besides FoxP3 or memory phenotype have all been referred to as “effector Tregs” in previous literature (29, 72). Tregs can inhibit immune responses by killing effector immune cells *via* release of granzymes and perforin as well as Fas-FasL interactions in normal and tumor settings (29). Tregs that express characteristics of Th effector cells and Tregs that harbor memory phenotypes have also been described in both settings (73).

Conventional CD4^+^CD127^lo^FoxP3^+^ Tregs and unconventional CD4^+^CD127^hi^FoxP3^+^ Tregs expressing cytotoxicity markers CD107 and FasL have been described in lymphoma and CLL patients (73). This study demonstrated that isolated CLL-derived Tregs killed CLL cells in *ex vivo* assays, indicating the potential to eliminate malignant cells (73). CLL-derived Tregs can switch toward effector Th phenotypes characterized by altered transcriptional and cytokine profiles when compared to healthy donors (74). These effector Tregs co-expressed FoxP3 and T-bet, GATA-3 or RORγt transcription factors resulting in Th1-like, Th2-like or Th17-like Tregs respectively. Th-like Treg subsets were increased in CLL patients compared to healthy donors (74). Other studies have similarly identified Tregs with characteristics of Th cells (75, 76).

It has been proposed that memory Treg differentiation might be a response to inflammatory factors in the TME (77). Chronic antigen stimulation and inflammation occurring in lymphoma and CLL may therefore potentially contribute to the presence of memory Tregs (78). Many investigators studying T-cell subsets in lymphoma and CLL have utilized variations of the widely accepted CD4^+^CD25^hi^CD127^-/lo^FoxP3^+^ flow cytometry gating strategy to identify Tregs, where CD127^+/hi^ cells representing memory T cells are excluded. However, Biancotto et al. reported that CD4^+^CD25^+^FoxP3^+^ Tregs in CLL patient PBMCs could be further divided according to canonical memory surface markers (79). Tregs were identified as naïve (CD45RA^+^), central memory (CD45RA^-^CD27^+^CCR7^+^), effector memory (CD45RA^-^CD27^+^CCR7^-^) and effector (CD45RA^-^CD27^-^CCR7^-^). The frequencies of these subsets were altered in CLL patients compared to healthy donor controls. CD39 is expressed on exhausted CD8^+^ T cells and mediates suppressive activity in Tregs (80, 81). CD39^+^CCR7^+^ Tregs were identified in CLL patients that were not found in healthy donors, indicating a memory Treg subset with high suppressive capacity (79). The study confirmed that these Tregs suppressed responder cell activity *ex vivo*. A separate study similarly found intratumoral CD27^+^CD45RA^-^ memory Tregs in FL patient samples (55).

CD8^+^ Tregs that express FoxP3 and exhibit suppressive function have been identified, but research on this rare subset is limited (82). CD8^+^CD25^+^FoxP3^+^ Tregs have been noted to be increased in CLL patients compared to healthy donors (79) as well as in progressive versus indolent CLL patients (83). Taken together, these studies suggest that Tregs harboring effector phenotypes are altered in lymphoma and CLL and exhibit variable function. Unconventional CD8^+^FoxP3^+^ cells are also altered in CLL, but their existence has not been described in lymphoma. The role of CD8^+^FoxP3^+^ cells in normal and tumor settings remains to be established.

## Expression of inhibitory receptors and immune checkpoint blockade

Tregs canonically express inhibitory surface receptors that mediate suppression including TIM-3, CTLA-4, TIGIT, LAG-3, PD-1 and NRP-1, among others. Elevated expression of these markers identifies more stable and functional Tregs (84). Earlier correlative studies indicated that CTLA-4^+^ and TIM-3^+^ Tregs associated with poorer outcomes in lymphoma patients (85, 86). These findings contradict other studies summarized above that relied on FoxP3 staining alone to identify Tregs. The role of inhibitory receptors, also referred to as immune checkpoints, in T-cell exhaustion has been widely acknowledged (87), and immune checkpoint blockade (ICB) is being tested in lymphoid malignancies (13). The impact of ICB on Tregs in these diseases has therefore become relevant. In this section, we summarize studies addressing the role of Tregs characterized by inhibitory receptor expression and discuss effects of ICB.

TIGIT is frequently expressed by Tregs in FL patients (88). Imbalance of TIGIT and CD226, its competitive co-stimulatory receptor, on CD8^+^ T cells has been concurrently noted (88). TIGIT^+^ Tregs are therefore likely to contribute to immunosuppression in FL. Another study found that LAG-3 was highly expressed in DLBCL patient derived-Tregs (89). In these patients, the majority of LAG-3^+^ Tregs co-expressed PD-1 and TIM-3. These Tregs could be divided into nTregs (defined as CD4^+^CD25^hi^CD127^lo^) and iTregs (defined as CD4^+^CD25^lo^CD127^lo^). This study implied that LAG3^+^ Tregs are involved in complex cellular interactions in the DLBCL microenvironment and suggested that combination PD-1/LAG-3 blockade warrants further investigation (89).

Mouse models have proven useful for examining inhibitory receptor expression on Tregs during disease progression and identifying ICB candidates. In a study utilizing the λ-MYC mouse model of BL, tumor-promoting Tregs were found to express canonical nTregs markers Helios and NRP-1 and recognized antigens expressed by lymphoma cells (71). The same group later observed that Tregs progressively upregulated suppressive phenotypic and functional markers during disease progression in this model, including FoxP3, CD25, CTLA-4, PD-1 and IL-10 (90). Lymphoma-derived Tregs limited responder cell proliferation and IFN-γ production more than WT-derived Tregs, and this suppressive ability was dependent on surface NRP-1, PD-L1 and soluble IL-10. The authors further asked whether ICB could affect Tregs. Combination PD-1/CTLA-4 blockade downregulated FoxP3 and CD25 expression on Tregs, indicating functional disruptions (90). These findings confirmed that Treg changes occurring during lymphoma development could be partially reversed by combination ICB in the λ-MYC model.

CTLA-4 (68) and LAG-3 (69) expression have been observed on Tregs in the Eμ-TCL1 mouse model of CLL that were found to exhibit tumor-promoting characteristics. Through unbiased clustering analysis of high dimensional flow cytometry data, another study found three previously undescribed clusters of Tregs in this model (1) KLRG1^+^CD69^+^ Tregs described as terminally differentiated, tumor-infiltrating Tregs (2) FoxP3^+^CD25^lo^LAG-3^+^ Tregs that were enriched in leukemic mice and represented a reservoir of suppressive Tregs (3) CD44^lo^CD62L^+^CD38^-^KLRG1^-^ Tregs that were not enriched in leukemic mice and represented naïve Tregs (91). KLRG1 has been proposed as an immune checkpoint and KLRG1^+^ Tregs have been described by other investigators (92, 93). However, KLRG1^+^ Treg function in the CLL microenvironment is not yet understood. Dual anti-PD-1/LAG-3 treatment restored an immunocompetent response with fewer Treg cells in this model (91). It is unclear if Tregs were directly affected or reduced as a secondary effect of leukemia clearance.

CD38 is expressed on some CLL cells and is a prognostic marker for CLL patients (94). CD38 is also expressed on Tregs and associated with suppressive activity (95). A higher proportion of CD38^+^ Tregs has been identified in CLL patients compared to healthy donors (59). This study found that CD38^+^ Tregs were positively associated with CD38^+^ CLL cells. CD38 blockade *ex vivo* directly induced cell death in CLL Tregs. Further, daratumumab (anti-CD38) systemic treatment reduced Tregs and expanded antitumor T-cell subsets in a patient-derived xenograft (PDX) CLL mouse model, suggesting that daratumumab provoked a potent antitumor response (59). Collectively, the studies reviewed here imply that ICB can affect Treg subsets that are altered in the context of lymphoma and CLL to enhance antitumor benefit. Blockade of novel checkpoints and combination approaches to modulate Tregs should therefore be investigated.

## T follicular regulatory cells

Tfh cells are required in the germinal center reaction where they assist B cells during memory differentiation and generation of high-affinity antibodies (22). T follicular regulatory (Tfr) cells develop from conventional Treg precursors or naïve CD4^+^ T cells and control germinal center B-cell responses by limiting Tfh function. Tfr cells express markers in common with Tfh cells such as BCL-6, PD-1, CXCR5 and ICOS as well as markers in common with conventional Tregs such as FoxP3, CTLA-4 and GITR (96).

Tfr cells have been identified in FL, DLBCL, and most recently, CLL patients. ICOS^+^CXCR5^+^FoxP3^+^ cells that expressed CTLA-4, GITR and Ki67, consistent with Tfr phenotype, were found to be increased in FL patient tissues compared to controls (55). Elevated Tfr characterized as CD4^+^CXCR5^+^FoxP3^+^ were found in (1) lower stage DLBCL patients compared to higher stage patients and (2) in DLBCL patients who remained in remission following chemoimmunotherapy compared to those who relapsed (97). In *ex vivo* experiments, stimulated Tfr expressed high levels of anti-apoptotic BCL-6, as well as IL-10 and TGF-β1. Tregs exhibited superior suppressive activity against responder T cells compared to Tfr. Interestingly, Tregs also provided superior support for tumor cell proliferation in coculture assays (97). This study therefore concluded that Tfr were functionally distinct from Tregs.

Another recent study demonstrated that CD4^+^CXCR5^+^ T cells from DLBCL patients could be divided into Tfr or Tfh based on whether they were CD25^+^ or CD25^-^ respectively (98). Tregs and Tfr differed in expression of transcription factors and cytokines, supporting the notion that they exert different functions. This study further described a mechanism whereby IL-21 secreted by Tfh regulated the expression of FoxP3 and IL-10 in Tfr in DLBCL patient samples (98).

Tfr has also recently been investigated in CLL patients, but little is known about their role in CLL. Nurse-like cells represent tumor-associated macrophages in CLL that support CLL survival and proliferation (99). Interestingly, coculture of nurse-like cells with CLL T cells increased Tfr percent and elevated expression of activation and proliferation markers including CD69, PD-1, CTLA-4, TGF-β, IL-10 and Ki67 (98). Collectively these studies indicate that Tfr in lymphoma and CLL are functionally distinct from Tregs. Further studies will be necessary to specifically determine associations of Tfr with clinical outcomes in FL and CLL patients.

## T regulatory type 1 cells

T regulatory type 1 (Tr1) cells have been described in other diseases besides lymphoid malignancies and are distinct from CD4^+^CD25^hi^FoxP3^+^ Tregs as they are not characterized by constitutive FoxP3 expression. Tr1 cells produce IL-10 and express coinhibitory receptors, but also harbor cytotoxic activity (100). A single study thus far has investigated Tr1-like cells in CLL and lymphoma (101). The authors observed accumulation of Tr1-like cells in CLL and DLBCL patients’ lymph nodes compared to reactive lymph node controls. Tr1-like cells were identified as CD4^+^EOMES^+^PD-1^+^ T cells expressing IL-10, as well as activation and cytotoxicity markers. In the transgenic and adoptive transfer Eμ-TCL1 mouse models of CLL, Tr1-like cells were found to be essential for CD4^+^ T cell-mediated control of leukemia development. Further, the study showed that IL-10 signaling was important for the cytotoxic capabilities of these cells (101). As Tr1 cells share phenotypic and functional similarities with both conventional Tregs and effector Tregs, further studies will be essential to define characteristics of this enigmatic subset in the context of lymphoid malignancies.

## Impact of targeted therapies

Targeted therapies have emerged as promising alternative treatments options to standard chemoimmunotherapy for B-NHL patients (102). Currently, approved targeted therapies include the immunomodulatory drug (iMiD) lenalidomide, several BTK inhibitors, BCL-2 inhibitor venetoclax, and several PI3K inhibitors (102). Although these inhibitors primarily target malignant B cells, they can directly modulate other immune cells, including T cells, due to cellular homology and off-target activity (103). T-cell modulation by targeted therapies can also occur indirectly due to malignant cell clearance and subsequent restoration of immune competency (103). ICB has demonstrated notable efficacy in several tumor types, including classical Hodgkin’s lymphoma (104). However, studies investigating ICB therapy in B-NHL patients have not yielded similar response rates thus far. The expression of multiple inhibitory receptors on Tregs in B-NHL (discussed in section 5) implies that Tregs could be affected by ICB. The impact of targeted therapies on Tregs has now become a topic of interest. Effects of targeted therapies on Tregs may be beneficial, for example, they may augment antitumor immunity. On the other hand, they can result in T-cell subset imbalance and contribute toward unwanted immune-mediated toxicities. In this section, we discuss studies that describe Treg modulation by approved targeted therapies (**Table 2**).

**Table 2:** Impact of targeted therapies on Tregs in patients with B-cell lymphoid malignancies.

Targeted therapies	Disease	Impact on Tregs	Reference
lenalidomide	CLL	decreased Tregs	(105)
lenalidomide/alemtuzumab	CLL	decreased Tregs	(106)
lenalidomide/obinutuzumab	FL	no effect on Treg suppressive activity	(107)
ibrutinib	CLL	decreased Tregs	(108–110)
acalabrutinib	CLL	decreased Tregs (trend)	(108)
zanubrutinib	CLL	decreased Tregs and lowered CTLA-4 expression on Tregs	(111)
venetoclax/obinutuzumab	CLL	decreased Tregs	(112)
venetoclax/ibrutinib	CLL	decreased Tregs	(112)
idelalisib	CLL	reduced Treg differentiation and function	(113, 114)
duvelisib/FCR	CLL	increased granzyme-B+ Tregs	(114)
rituximab/ipilimumab	B-NHL	decreased Tregs and CTLA-4+ Tregs	(115)

Lenalidomide has demonstrated favorable clinical outcomes as a single agent or in combinations, eliciting durable responses in B-NHL (116). Lenalidomide is currently approved for relapsed MCL patients who have progressed after two prior therapies, previously treated MZL and FL patients in combination with rituximab, and R/R DLBCL patients in combination with tafasitamab (116). Prior studies revealed that lenalidomide exerts immunomodulatory effects on CLL and FL T cells (19, 20). Immunophenotyping analysis of clinical trial samples demonstrated Treg modulation after lenalidomide treatment. In CLL patients, Treg percent and number were diminished after lenalidomide single agent treatment comparable to levels in healthy controls (105). Similar results were observed in CLL patients treated with lenalidomide in combination with alemtuzumab (106). In FL patients, lenalidomide treatment triggered T-cell proliferation in PBMCs without compromising Treg suppressive activity (107). Further, a pre-existing Treg signature indicated inferior response to treatment (107). The overall trend suggests that Treg distribution but not function is changed in patients treated with lenalidomide.

Ibrutinib is an irreversible BTK/ITK inhibitor that is currently approved for patients with MCL who have received at least one prior therapy, CLL/SLL including those with 17p deletion, MZL who have received at least one prior anti-CD20 therapy, and Waldenstrom’s macroglobulinemia (WM) (67). Both BTK and ITK belong to the TEC family of kinases (117). While BTK is expressed in B cells and crucial for B-cell receptor signaling (102), ITK is expressed in T cells and necessary for control of TH differentiation (118), including Treg/Th17 balance (119). Accordingly, ibrutinib directly regulates T cells *via* ITK inhibition (120). Acalabrutinib is a second-generation, irreversible BTK inhibitor with minimal off-target kinase activity against Tec-family kinases compared to ibrutinib that is currently approved for patients with MCL and CLL/SLL (121).

T-cell composition analysis of CLL patients treated with ibrutinib or acalabrutinib demonstrated that ibrutinib exerted immunomodulatory effects on T cells. These effects occurred *via* inhibition of ITK-mediated, activation-induced cell death (108). Ibrutinib-treated patients harbored reduced Treg to CD4+ ratio compared to baseline, and this occurred due to selective expansion of non-Treg CD4+ subsets. Although acalabrutinib-treated patients also showed a trend in reduced Treg to CD4+ ratio compared to baseline, these differences were not significant (108). Other studies similarly reported lower Treg percent in ibrutinib-treated CLL patients in both first-line and R/R settings (109, 110). Zanubrutinib, another second-generation irreversible BTK inhibitor that is approved for R/R MZL patients, reduced Treg percent and CTLA-4 expression on Tregs in R/R CLL patients (111). Data addressing the effect of BTK inhibitors on Tregs in patients with other types of lymphoid malignancies besides CLL have not yet been reported.

Venetoclax is a selective inhibitor of the anti-apoptotic protein BCL-2 that is currently approved for patients with CLL, and is being investigated for patients with R/R FL, MZL and WM as a single agent or in combination with anti-CD20 antibody (67). BCL-2 expression is constitutively upregulated in several types of B-NHL, conferring anti-apoptotic signals and promoting malignant cell survival (103). T cells also express and depend on BCL-2, but this dependence varies among T-cell subsets (103). As mentioned above, a recent study implied that BCL-2 could be important for Treg function in CLL patients (66). Immune composition was altered in CLL patients after venetoclax treatment compared to baseline (112). Specifically, patients treated with venetoclax/obinutuzumab (anti-CD20) or venetoclax/ibrutinib harbored reduced frequencies of Tregs compared to baseline (112).

The PI3K/AKT pathway is essential for malignant B-cell survival, proliferation, and function (122). PI3K inhibitors targeting one or more class I catalytic subunits (p110α, β, γ, δ) abrogate pro-survival signaling in B cells. Several PI3K inhibitors have been approved for use in B-NHL patients including idelalisib (targeting δ), duvelisib (targeting δ/γ) and copanlisib (targeting α/δ) (123). As T cells also depend on PI3K signaling for differentiation and function (124), the effects of PI3K inhibitors on T cells have been investigated. CLL patients treated with idelalisib exhibit reduced Treg differentiation and function (113, 114). *Ex vivo* treatment of CLL T cells with idelalisib disrupted Treg function, implying that PI3K inhibitors directly target Tregs (125). Genetic ablation of p110δ or treatment with PI3K inhibitors in the Eμ-TCL1 mouse model of CLL corroborated these findings (126–128).

Treg disruption by PI3K inhibitors has been associated with frequent and severe immune-mediated adverse events (67, 129). A noteworthy study also found that low baseline Tregs in patients treated with duvelisib/FCR predicted immune-mediated toxicities (130). The authors observed that granzyme B+ Tregs were increased after duvelisib/FCR treatment in CLL patients and suggested that this phenotype indicated more activated and apoptotic Tregs (130). PI3K inhibitors are now being developed with strategies in mind to mitigate adverse events by limiting Treg-depleting effects (131, 132). On another note, combinations with PI3K inhibitors are being explored due to their potential ability to restore immune competence. Idelalisib treatment hampered recruitment of Tregs to the FL microenvironment and unleased the activity of venetoclax, indicating potential utility of combining PI3K inhibition with venetoclax (133).

ICB is currently being investigated for treatment of B-NHL. Many trials have focused on antibodies blocking PD-1/PD-L1 and CTLA-4 as single agents or in combinations in B-NHL patients (134). Ipilimumab is an anti-CTLA-4 antibody that is approved to treat metastatic melanoma and several other tumor types (135). Studies in patients with solid tumors indicated that ipilimumab treatment depleted intratumoral Tregs possibly through antibody-dependent cell mediated cytotoxicity (136). Ipilimumab has been tested in patients with R/R B-NHL as a single agent (137), in combination with nivolumab (anti-PD-1) (138), and in combination with rituximab (115), but these treatments elicited modest efficacy rates. Correlative analysis of B-NHL patients treated with rituximab/ipilimumab demonstrated that, after an initial expansion, peripheral blood Tregs and CTLA-4^+^ Tregs generally decreased over time (115). Additionally, CD45RA^-^ Treg to Treg ratio was elevated in responders compared to non-responders at baseline and following therapy. This data indicated that CD45^-^ Treg to Treg ratio could potentially identify patients who would respond to this combination, however this would need to be validated in larger cohorts (115). Whether antitumor efficacy elicited by ipilimumab in B-NHL patients depends on Treg modulation remains to be further explored.

Taken together, these studies reveal that Tregs are consistently reduced in patients treated with the targeted inhibitors mentioned above. Since Tregs express targets such as ITK, PI3K catalytic subunits and BCL-2, some effects may be direct. Evidence also suggests that Tregs may be affected by ICB therapy. Lower Treg levels could beneficially facilitate restoration of antitumor T-cell responses. In the case of PI3K inhibitors, reduced Tregs appear to associate with increased immune-mediated toxicities, which has hampered the development of this class of inhibitors.

## Conclusions and future perspectives

Tregs are key players in the microenvironment of lymphoid malignancies. In this review, we highlighted studies exploring Treg prognostic value, origin, phenotype, function, and the impact of novel therapies. While elevated Tregs indicated better outcomes in FL and DLBCL patients, the opposite trend was observed in CLL patients. Similarly, there are conflicting reports regarding prognostic value of Tregs in solid cancers. While studies in melanoma, lung, ovarian and gastric cancers demonstrated that higher Treg levels in the tumor microenvironment generally correlated with worse prognosis, another study showed that Tregs were correlated with better survival in head and neck, colorectal and esophageal cancers (139). One caveat of studies utilizing FL and DLBCL biopsy specimens is that it is difficult to distinguish Tregs from Tfr cells by immunohistochemistry using FoxP3 staining alone. Inhibitory receptors and CXCR5 could be useful to distinguish these two populations. Further correlative studies to determine if Tregs predict response to novel therapeutics such as targeted therapies, ICB and CAR T-cell therapies in B-NHL patients will be noteworthy.

The studies reviewed here revealed that Tregs can be induced and recruited to the TME, a process facilitated by dysregulated proteins expressed by malignant B cells. Tregs and Tfr are also modulated by other immune subsets in the TME like MDSCs and Tfh, respectively. Tregs comprise phenotypically heterogenous subsets and exhibit features of plasticity. Overall trends suggest that Tregs exert tumor-promoting roles in lymphoma and CLL. Similarly, studies in other cancer types have found that Tregs in the TME exhibit phenotypic heterogeneity and that TME signals can affect Treg differentiation, proliferation and immunosuppressive activity (140). Mouse model studies to further examine Treg stability, function, and response to microenvironmental cues during disease progression could provide deeper insight in the context of lymphoid malignancies.

Tregs expressing increased levels of inhibitory receptors in lymphoma and CLL may be targeted by ICB. As ICB approaches have produced subpar responses in these patients, it would be appealing to determine whether Treg disruption could improve efficacy. In addition, Treg studies could reveal new target candidates and rationale for combinatorial therapies. In contrast, targeted agents have been largely successful in terms of efficacy and tolerability, and therefore they have been incorporated into treatment regimens for lymphoma and CLL patients. Clinical correlatives demonstrated that Tregs are reduced in patients treated with ibrutinib, venetoclax, PI3K inhibitors and ipilimumab. Mouse model studies supported these observations. In studies of patients with other cancer types, reduction of Tregs in tumor tissues have been strongly correlated with clinical efficacy of ipilimumab. In patients with lymphoid malignancies treated with PI3K inhibitors, loss of Tregs have been associated with occurrence of immune-mediated adverse events. Due to expression of molecular targets in Tregs it is likely that these agents exhibit direct immunomodulatory effects on Tregs. However, Treg decrease could also depend on malignant cell clearance. Studies in other tumor types have demonstrated that PI3K inactivation by genetic ablation or pharmacological inhibition disrupts Treg function and Treg-mediated immune tolerance to cancer (141, 142). Functional studies will be required to comprehend the effects of targeted agents on Treg function in the context of lymphoid malignancies.

In the future, it would be interesting to explore optimal methods to modulate Tregs – to strike a balance between restoring antitumor immunity while preventing immune-mediated adverse events. Since most studies characterizing the effects of targeted agents on Tregs were performed in CLL patients, studies on patients with other lymphoid malignancies would enrich our understanding of this area. Future developments could help to devise strategies to optimize therapeutic interventions such as combinatorial approaches with improved efficacy and safety.

## Author contributions

KM conceived, wrote, and revised the manuscript. AU wrote and revised the manuscript. ES and JP-I conceived and revised the manuscript. All authors contributed to the article and approved the submitted version.

## Funding

We would like to thank Jorge and Silvia Ferioli for the generous financial support they provided to help make this project possible.

## Acknowledgments

**Figure 1** was created with BioRender.com.

## Conflict of interest

JP-I declares consulting and fees from Janssen Pharmaceuticals, Pharmacyclics, AbbVie and AstraZeneca, and research funding from SecuraBio.

The remaining authors declare that the research was conducted in the absence of any commercial or financial relationships that could be construed as a potential conflict of interest.

## Publisher’s note

All claims expressed in this article are solely those of the authors and do not necessarily represent those of their affiliated organizations, or those of the publisher, the editors and the reviewers. Any product that may be evaluated in this article, or claim that may be made by its manufacturer, is not guaranteed or endorsed by the publisher.
